# Spatio-Temporal Gait Variable Differences Between Independent and Assisted Living Older Adults

**DOI:** 10.1093/geroni/igaa057.3234

**Published:** 2020-12-16

**Authors:** Christopher Nightingale, Jennifer McNulty, Stephen Butterfield

**Affiliations:** University of Maine, Orono, Maine, United States

## Abstract

As the US population continues to age, it is important to gain a better understanding of factors that differ between independently living older adults and those that require assisted living. Accidental falls contribute to decreased quality of life for many older adults, and considerable research has been conducted examining measures of fall-risk and potential interventions. In this pilot study, we investigated fall-risk via assessment of spatio-temporal variables associated with gait in independent and assisted-living older adults aged 65+. Two gender-matched groups of participants were tested on seven gait variables (step length, stride length, contact time, contact phase, foot flat, propulsive phase, and average speed) utilizing Opto-Gait technology. Each group included sixteen participants. Independent living participants were recruited through word of mouth and flyers at local senior centers. Assisted living participants all resided in one local health care facility and were recruited via word of mouth and flyers posted in the facility. A one way ANOVA with an alpha level of .05 revealed significant differences between groups in contact time, foot flat, propulsive phase, and average speed. The independent living group performed better on each variable. These findings indicate that older adults living in assisted living facilities may be at much greater risk of accidental fall than their independent living counterparts. While this study identified several key factors related to instability among old adults, additional investigation is warranted to further examine the relationships among gait, fall-risk, and differentiators between independently living and assisted living older adults in these variables.

